# Accelerated genome engineering of *Pseudomonas putida* by I‐*Sce*I―mediated recombination and CRISPR‐Cas9 counterselection

**DOI:** 10.1111/1751-7915.13396

**Published:** 2019-03-12

**Authors:** Nicolas T. Wirth, Ekaterina Kozaeva, Pablo I. Nikel

**Affiliations:** ^1^ The Novo Nordisk Foundation Center for Biosustainability Technical University of Denmark 2800 Kongens Lyngby Denmark

## Abstract

*Pseudomonas* species have become reliable platforms for bioproduction due to their capability to tolerate harsh conditions imposed by large‐scale bioprocesses and their remarkable resistance to diverse physicochemical stresses. The last few years have brought forth a variety of synthetic biology tools for the genetic manipulation of pseudomonads, but most of them are either applicable only to obtain certain types of mutations, lack efficiency, or are not easily accessible to be used in different *Pseudomonas* species (e.g. natural isolates). In this work, we describe a versatile, robust and user‐friendly procedure that facilitates virtually any kind of genomic manipulation in *Pseudomonas* species in 3–5 days. The protocol presented here is based on DNA recombination forced by double‐stranded DNA cuts (through the activity of the I‐*Sce*I homing meganuclease from yeast) followed by highly efficient counterselection of mutants (aided by a synthetic CRISPR‐Cas9 device). The individual parts of the genome engineering toolbox, tailored for knocking genes in and out, have been standardized to enable portability and easy exchange of functional gene modules as needed. The applicability of the procedure is illustrated both by eliminating selected genomic regions in the platform strain *P. putida* KT2440 (including difficult‐to‐delete genes) and by integrating different reporter genes (comprising novel variants of fluorescent proteins) into a defined landing site in the target chromosome.

## Graphical abstract

The present protocol describes a streamlined method for precise genome engineering of *Pseudomonas* species. By using a combination of fluorescent markers, coupled with the activity of the I‐SceI meganuclease or CRISPR/Cas9‐mediated counterselection, virtually any genomic manipulation can be performed in 3–5 days.

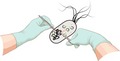

## Introduction

Supported by the advances in synthetic biology and metabolic engineering made in the last few years, several microbes have emerged from a rather shadowy existence to become suitable biotechnological platforms (Calero and Nikel, 2019). Several members of the genus *Pseudomonas*, for instance, are being adopted by a steadily increasing number of laboratories worldwide as *chassis* for both fundamental and applied studies (Loeschcke and Thies, 2015; Benedetti *et al*., 2016; Dvořák *et al*., 2017; Nikel and de Lorenzo, 2018). *Pseudomonas* species, usually found in natural environments, distinguish themselves from other microorganisms by an unrivalled wealth of biochemical functions that are embedded in robust and flexible metabolisms (Poblete‐Castro *et al*., 2017) – allowing bacteria to adapt to a diverse range of stressful conditions (Nikel *et al*., 2016). A rich set of synthetic biology tools has been developed to untap the metabolic potential of key representatives of the genus, e.g. *P. putida*,* P. taiwanensis* and *P. fluorescens* (Gomez *et al*., 2012; Domröse *et al*., 2017; Martínez‐García and de Lorenzo, 2017; Cook *et al*., 2018; Wynands *et al*., 2018; Volke *et al*., 2019). A breakthrough in the standardization of a toolbox of reliable molecular tools for Gram‐negative bacteria has been the creation of the *Standard European Vector Architecture* (SEVA) platform (Silva‐Rocha *et al*., 2013; Martínez‐García *et al*., 2015), including a variety of genetic parts (e.g. promoters, origins of replication and antibiotic‐resistance determinants) that are functional in a number of *Pseudomonas* species. More recently, synthetic libraries of constitutive promoters have facilitated the predictable expression of a gene of interest with a desired strength (Elmore *et al*., 2017), particularly when the promoter of choice is to be combined with standardized translational couplers (Zobel *et al*., 2015).

Considerable efforts in the scientific *Pseudomonas* community have led to the development of tools for the construction of clean deletion mutants. Counterselection strategies adapted to *Pseudomonas* include a *pyrF*‐based dual‐selection system (Galvão and de Lorenzo, 2005), *sacB* as counterselectable marker (Gay *et al*., 1985) and the *upp* gene (uracil phosphoribosyltransferase) in an *upp‐*deficient mutant (Graf and Altenbuchner, 2011). The λ *Red* system, widely used in *Escherichia coli* for recombineering (Murphy, 2016), has been combined with Cre/*loxP* site‐specific recombination to enable genomic modifications with linear DNA fragments (Luo *et al*., 2016). Cook *et al*. (2018) introduced a recombineering method based on the λ *Red* system together with CRISPR‐Cas9 counterselection. Such recombinase‐based genome‐editing approaches have recently been complemented by the design of a RecET system for *P*. *putida* (Choi *et al*., 2018; Choi and Lee, 2019), and Aparicio *et al*. (2018) further honed the CRISPR‐Cas9 counterselection strategy, combining it with Ssr‐based recombination for genome engineering. Among all these efforts, the standardized genome engineering method developed by Martínez‐García and de Lorenzo (2011), based on the chromosomal integration of a suicide plasmid followed by the action of the homing nuclease I‐*Sce*I from *Saccharomyces cerevisiae*, excels for its efficiency and robustness to mediate any kind of genetic modification (e.g. large deletions, point mutations and allelic exchanges) – and is thus amongst the most intensively used by the scientific *Pseudomonas* community. The system relies on vector pEMG; this vector contains the conditional origin of replication *R6K* (Table 1), which requires the cognate Rep protein π encoded by *pir* for replication. In bacterial strains that lack *pir*, vector pEMG behaves as a suicide plasmid. The vector contains a polylinker region (multiple cloning site) flanked by two I‐*Sce*I recognition sites cloned into the *lacZ*α fragment, thus allowing for blue–white screening in *E. coli* cells carrying the *lacZ*Δ*M15* mutation. A set of auxiliary SEVA plasmids harbours the gene encoding I‐*Sce*I under the control of the 3‐methylbenzoate (3‐*m*Bz)‐inducible XylS/*Pm* expression system from strain mt‐2 (Martínez‐García and de Lorenzo, 2011).

**Table 1:** Plasmids used in this work

Name^a^	Relevant features^b^	Source or reference
pEMG	Suicide vector used for deletions in Gram‐negative bacteria; *oriT*,* traJ*,* lacZ*α, *oriV(R6K)*; Km^R^	Martínez‐García and de Lorenzo (2011)
pRK600	Helper plasmid used for conjugation; *oriV(ColE1)*,* mob(RK2)*,* tra(RK2)*; Cm^R^	Kessler *et al*. (1992)
pGNW2	Derivative of vector pEMG carrying *P*_*14g*_→*msfGFP*	This work
pGNW4	Derivative of vector pGNW2; Sm^R^	This work
pGNW6	Derivative of vector pGNW2; Gm^R^	This work
pGNW2**·**Δ*nicX*	Derivative of vector pGNW2 carrying HRs to delete *nicX* (*PP_3945*) in *P. putida* KT2440	This work
pGNW4**·**Δ*nicX*	Derivative of vector pGNW4 carrying HRs to delete *nicX* (*PP_3945*) in *P. putida* KT2440	This work
pGNW6**·**Δ*nicX*	Derivative of vector pGNW6 carrying HRs to delete *nicX* (*PP_3945*) in *P. putida* KT2440	This work
pGNW2**·**Δ*aceEF*	Derivative of vector pGNW2 carrying HRs to delete *aceEF* (*PP_0338‐PP_0339*) in *P. putida* KT2440	This work
pSEVA2313	Expression vector; *oriV(pBBR1)*,* P*_*EM7*_; Km^R^	This work
pS2313**·**R	Derivative of vector pSEVA2313 carrying *P*_*EM7*_→*mRFP1* (Campbell *et al*., 2002); Km^R^	This work
pS2313**·**B	Derivative of vector pSEVA2313 carrying *P*_*EM7*_→*mBFP2* (Subach *et al*., 2011); Km^R^	This work
pS2313**·**O	Derivative of vector pSEVA2313 carrying *P*_*EM7*_→*mOrange2* (Shaner *et al*., 2008); Km^R^	This work
pS2313**·**T	Derivative of vector pSEVA2313 carrying *P*_*EM7*_→*mTurquoise2* (Goedhart *et al*., 2012); Km^R^	This work
pGNW2**·**LP::*P*_*EM7*_‐mRFP	Derivative of vector pGNW2 carrying *P*_*EM7*_→*mRFP1*	This work
pGNW2**·**LPR	Derivative of vector pGNW2 carrying HRs to insert *P*_*14g*_(*BCD2*)→ *mRFP1* into a landing pad in the chromosome of *P. putida* KT2440	This work
pGNW2**·**LPG	Derivative of vector pGNW2 carrying HRs to insert *P*_*14g*_(*BCD2*)→ *msfGFP* into a landing pad in the chromosome of *P. putida* KT2440	This work
pGNW2**·**LPB	Derivative of vector pGNW2 carrying HRs to insert *P*_*14g*_(*BCD2*)→*mBFP2* into a landing pad in the chromosome of *P. putida* KT2440	This work
pGNW4**·**LPO	Derivative of vector pGNW4 carrying HRs to insert *P*_*14g*_(*BCD2*)→ *mOrange2* into a landing pad in the chromosome of *P. putida* KT2440	This work
pGNW6**·**LPT	Derivative of vector pGNW6 carrying HRs to insert *P*_*14g*_(*BCD2*)→ *mTurquoise2* into a landing pad in the chromosome of *P. putida* KT2440	This work
pSEVA128S	Helper plasmid; *oriV(RK2)*,* xylS*,* Pm*→*I‐SceI*; Amp^R^	Aparicio *et al*. (2015)
pSEVA228S	Helper plasmid; *oriV(RK2)*,* xylS*,* Pm*→*I‐SceI*; Km^R^	Aparicio *et al*. (2015)
pSEVA428S	Helper plasmid; *oriV(RK2)*,* xylS*,* Pm*→*I‐SceI*; Sm^R^	Aparicio *et al*. (2015)
pSEVA628S	Helper plasmid; *oriV(RK2)*,* xylS*,* Pm*→*I‐SceI*; Gm^R^	Aparicio *et al*. (2015)
pSEVA1213S	Helper plasmid; *oriV(RK2)*,* P*_*EM7*_→*I‐SceI*; Amp^R^	This work
pSEVA6213S	Helper plasmid; *oriV(RK2)*,* P*_*EM7*_→*I‐SceI*; Gm^R^	This work
pSEVA448	Expression vector; *oriV(pRO1600/ColE1)*,* xylS, Pm*; Sm^R^	Silva‐Rocha *et al*. (2013)
pSEVA421.Cas9tr	Cloning vector; *oriV(RK2)*,* cas9*,* tracr*RNA; Sm^R^	Aparicio *et al*. (2018)
pS448**·**CsR	Derivative of vector pSEVA448 used for CRISPR‐Cas9 counterselection; *xylS* (cured of *Bsa*I restriction sites), *Pm*→*cas9*,* P*_*EM7*_→*sgRNA*; Sm^R^	This work
pS448**·**CsR_*aceEF*	Derivative of vector pS448**·**CsR carrying *P*_*EM7*_→*aceF‐*targeting *sgRNA*; Sm^R^	This work

**a.** Plasmids can be obtained from Addgene (http://www.addgene.org) with the following deposit numbers: pGNW2 (122086), pGNW4 (122088), pGNW6 (122093), pSEVA1213S (122095), pSEVA6213S (122094) and pS448**·**CsR (122096).

**b.** Antibiotic markers: *Amp*, ampicillin; *Cm*, chloramphenicol; *Km*, kanamycin; *Sm*, streptomycin; and *Gm*, gentamicin. HRs, homology regions.

In this study, we present a smooth and quick workflow for genome editing of *P*. *putida* based on the action of I‐*Sce*I or CRISPR/Cas9 as a counterselection strategy. The integration of genes encoding fluorescent proteins (and antibiotic‐resistance determinants) into the target locus circumvents experimental steps that would be needed to ensure the presence of the suicide vector. Furthermore, we propose the adoption of the *USER* assembly method (Smith *et al*., 1993; Bitinaite and Nichols, 2009; Nour‐Eldin *et al*., 2010; Cavaleiro *et al*., 2015) to enable the quick and highly accurate assembling of homology regions (HRs) required for the genome engineering protocol. Helper plasmids for the constitutive expression of the gene encoding the homing I‐*Sce*I endonuclease from yeast were also designed to avoid the addition of chemical inducers during the process. The resulting streamlined protocol enables the introduction of virtually any genomic modification in *P*. *putida* within 5 days (for a single genetic manipulation including the construction of all necessary plasmids) down to 3 days (time required for each mutagenesis round once the relevant plasmids have been constructed), followed by plasmid curing. We demonstrate the potential of the technique by deleting both non‐essential and difficult‐to‐knock‐out metabolic genes in the platform strain KT2440. We furthermore show the integration of genes encoding different fluorescent proteins into a suitable landing pad in the *P. putida* chromosome and discuss their application as insertional reporters.

## Technical implementation

**Figure 1:**
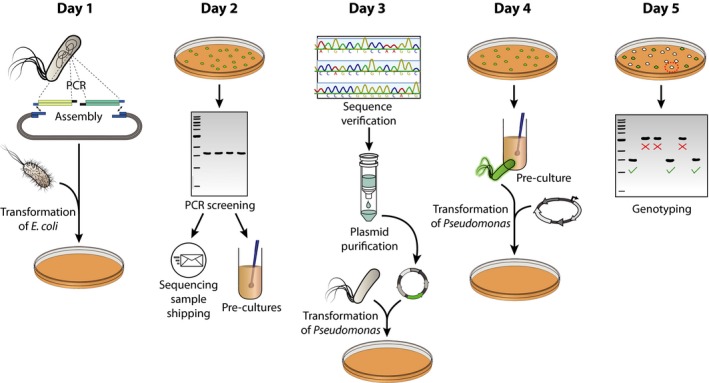
Overview diagram of the genome engineering procedure in *Pseudomonas putida*. On the delivery date of the designed oligonucleotides, homologous regions are amplified from the *Pseudomonas* genome and assembled into a suitable pGNW vector. The resulting plasmid is then delivered into *E*. *coli* λ*pir* cells *via* chemical transformation. Furthermore (if applicable), a synthetic spacer, completing a specific synthetic guide RNA (sgRNA), is separately prepared and cloned into the CRISPR‐Cas9―vector pS448**·**CsR. On day 2, *E*. *coli* transformants are screened for the correct pGNW insert size (and spacer insertion in vector pS448**·**CsR, if applicable) *via* colony PCR, the resulting amplicons are sent out for sequencing, and the corresponding clones are used to inoculate liquid cultures. Day 3 includes the verification of sequence integrity of the insert in the pGNW vector, the purification of individual plasmids, and their delivery into *Pseudomonas* either *via* electroporation or tri‐parental mating. The resulting co‐integrants are enriched in liquid LB cultures during the course of day 4 and transformed with either an I‐*Sce*I―bearing plasmid or plasmid pS448**·**CsR carrying an appropriate sgRNA. Finally, *Pseudomonas* colonies without fluorescence are tested on day 5 for their genotype *via* colony PCR.

The overall genome‐editing procedure begins with the construction of donor DNA that is cloned into the suicide vector pGNW and derivatives thereof (Fig. 1). Such donor DNA, containing suitable HRs, serves as template for the endogenous homologous DNA recombination machinery. The procedure also includes the cloning of a target‐specific synthetic guide RNA (sgRNA) into a dedicated CRISPR‐Cas9 plasmid (when applicable), the integration of the donor DNA into the *Pseudomonas* chromosome, and the specific cleavage of chromosomal DNA by endonucleases (i.e. I‐*Sce*I or Cas9) for the resolution of co‐integrates. The specific steps of the procedure are detailed in the sections below.

### Design and construction of plasmids

#### Construction of the suicide vector pGNW and derivatives

We first equipped plasmid pEMG with the gene encoding the monomeric superfolder GFP [msfGFP (Landgraf, 2012)] in the vector backbone while screening different combinations of promoters and translation initiation regions (*TIR*) that would give a high fluorescence output when the plasmid is integrated into the target chromosome. The fusion of the strong constitutive *P*_*14g*_ promoter and a variation of the *TIR* sequence preceding *msfGFP* (Zobel *et al*., 2015) allowed for the direct visualization of *Pseudomonas* colonies harbouring a single (chromosomal) copy of the novel vector pGNW2 (Table 1) by inspecting the plates on a blue‐light transilluminator. A set of pGNW vectors was constructed by swapping the kanamycin resistance (Km^R^) determinant present in vector pGNW2 by the corresponding, SEVA‐based genes conferring streptomycin (Sm^R^) or gentamicin (Gm^R^) resistance, giving rise to vectors pGNW4 and pGNW6, respectively (Table 1).

**Figure 2:**
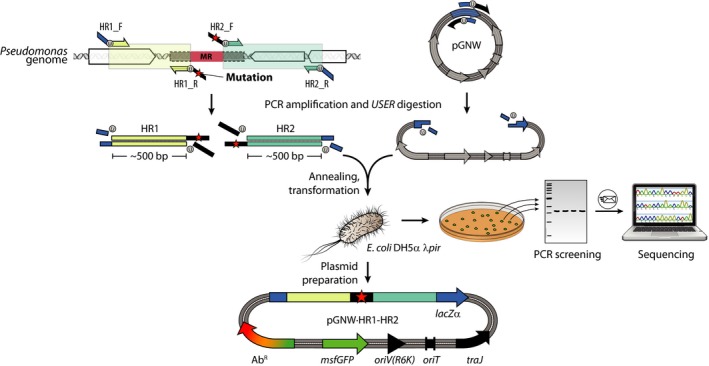
Workflow for the construction of derivatives of pGNW vectors for genome engineering. Two homology regions (HR1 and HR2), each one spanning about 500 bp and located upstream and downstream of the mutagenesis region (MR), are amplified from genomic DNA of *Pseudomonas via* PCR. Modifications (i.e. insertions or substitutions) are introduced between the HRs as overhangs in the oligonucleotides (indicated in the diagram with a red star) or as additional DNA fragments (not shown). For gene deletions, the sequences of the HRs are designed so that they frame the genome sequence to be deleted. HR1 and HR2 are fused and integrated into one of the pGNW vectors *via USER* assembly (shown here) or alternative molecular cloning techniques. The thereby assembled pGNW plasmid is then introduced into *E*.**** *coli* DH5α λ*pir*. Individual *E*. *coli* clones obtained after transformation are examined for green fluorescence on a blue‐light transilluminator and checked for the correct pGNW insert size *via* colony PCR, and the purified amplicon is sent for sequencing. After the sequence integrity is confirmed, pGNW plasmids are purified from the respective *E. coli* strain and saved for the next step.

A pGNW plasmid ready for genome engineering in *Pseudomonas* (Fig. 2) is composed of the vector backbone with an antibiotic‐resistance gene, the R6K origin of replication [*oriV(R6K*)], the *traJ* gene and a relaxation region (*oriT*) required for bacterial conjugation, and a site‐specific DNA insert that (i) is homologous to the target locus (i.e. HRs upstream and downstream to the region of interest) within the *Pseudomonas* genome and (ii) contains a desired modification. Such modification can be as short as a single base for a point mutation or arbitrarily long for the insertion of whole gene clusters. The insert and the vector backbone are assembled employing standard protocols, e.g. traditional cloning by restriction digest and ligation (Sambrook and Russell, 2001), Gibson assembly (Gibson *et al*., 2009) or *USER* assembly (Cavaleiro *et al*., 2015) that is recommended and further described here.

#### Oligonucleotide design

One primer pair is required to amplify each fragment that constitutes the pGNW derivative *via* PCR. Depending on the type of modification (see description below), this means that a minimum of three oligonucleotide pairs are required for cloning. A first pair of oligonucleotides (pGNW‐*USER*_F and pGNW‐*USER*_R, see Table S1) is used to reverse‐amplify the pGNW backbone, and the resulting linear fragment can be used in a standardized way for every cloning procedure. Two further pairs of primers are required to amplify the homologous regions HR1 and HR2 from the *Pseudomonas* genome, flanking the mutagenesis region (MR) targeted. The sequences of the primers HR1_F and HR2_R (Fig. 2) are determined by the MR to be modified (or deleted), and by the type of modification (i.e. deletion, insertion or substitution).

- Identify the ends of the MR sequence. The binding part of HR1_R starts upstream of the MR and extends into HR1, while the binding part of HR2_F starts downstream of the 3′‐end of the MR and extends into HR2 (Fig. 2). Extend the sequence of both oligonucleotides so that they fulfil general primer design requirements satisfactorily (i.e. 18–25 nt length, 40–60% GC content and GC‐clamp).
  - For deletions of chromosomal sequences, use the last ˜ 100 bp of HR1 and the first ˜ 100 bp of HR2 as input sequences for the *AMUSER* tool (Genee *et al*., 2015), available online at http://www.cbs.dtu.dk/services/AMUSER/, to identify suitable *USER* overhangs for the two primers. Add the required bases to the 5′‐end of both oligonucleotides. For gene deletions to have minimum polar effects and to avoid the potential creation of toxic, truncated polypeptides, we recommend leaving the *START* and *STOP* codons of the target gene intact and deleting the interjacent sequence. Upon deletion, this approach will leave e.g. 5′‐ATG TGA‐3′ in lieu of the coding sequence, where ATG and TGA are the *START* and the *STOP* codons, respectively.
  - For substitutions and small insertions, the mutation can be introduced within the primer overhangs. Add the desired sequence including the modification to the 5′‐end of HR2 and use the first ˜ 100 bp of this extended HR2, as well as the last ˜ 100 bp of HR1, as input sequences for the *AMUSER* online tool. Add any additional bases including the *USER* overhangs to the 5′‐end of both primers.
  - For larger insertions (exceeding 2× the annealing region of the primers), additional DNA fragments should be amplified separately and assembled with HR1 and HR2 to form the insert to be cloned in the pGNW vector of choice. The *AMUSER* tool can be used to design primers with suitable overhangs for their assembly.
- Design HR1_F at a distance of ˜ 500 bp upstream of HR1_R with a similar melting temperature. Design HR2_R at ˜ 500 bp downstream of HR2_F (Fig. 2). Add the motif 5′‐AGA TCC U‐3′ as the primer overhang to HR1_F, and 5′‐AGG TCG ACU‐3′ as overhang to HR2_R. These two overhangs match the ones that have been used in the primers to reverse‐amplify vector pGNW.
- Design one pair of ‘*g*‐check’ primers to test for the genotype of individual colonies after the genome engineering procedure. For deletions and insertions larger than 30 bp, the primers bind upstream and downstream of the MR, respectively, and amplify a product that can be distinguished from the wild‐type sequence with agarose gel electrophoresis according to its size (a size difference of > 10% is recommended). For substitutions of identical size, one primer should be able to bind only within the MR. The wild‐type sequence should thus give no amplification. For substitutions of only a few base pairs, the mutation‐containing region must be amplified and the genotype confirmed by restriction analysis (if applicable) or sequencing.

#### Amplification and assembly of DNA fragments

- Amplify vector pGNW using 5 ng of plasmid as template and primers pGNW‐*USER*_F and pGNW‐*USER*_R (Table S1) using *Phusion*™ U Hot Start DNA polymerase. Use standard reaction conditions as recommended by the manufacturer, with an elongation time of 3 min as well as an annealing temperature ‘touchdown’ from 65 to 59°C (Don *et al*., 1991), with a decrement of −1°C per cycle and a final annealing temperature of 58°C for 30 further cycles. We recommend to perform gel purification on the amplified pGNW fragment and to use the purified product as template for further PCRs. In this way, digestion with *Dpn*I to remove circular plasmids (i.e. template) can be omitted. We further recommend generating a large amount of linearized pGNW vector in several parallel PCRs for repeated use in *USER* assembly.
- Perform separate amplification reactions on purified genomic *Pseudomonas* DNA with the adequate primer pairs (HR1_F/HR1_R and HR2_F/HR2_R) to generate the HR1 and HR2 fragments. Perform, if needed, additional PCRs to generate the DNA fragments required for insertions. We generally recommend the use of a touchdown PCR protocol (Don *et al*., 1991) to reduce the formation of unspecific by‐products.
- Analyse a 3‐ to 5‐μl aliquot of each PCR by agarose gel electrophoresis to verify the correct amplification of the products. The concentrations of the fragments can be roughly estimated from the intensities of their bands. If agarose gel electrophoresis reveals the presence of non‐specific by‐products, the desired bands have to be purified from a gel prior to cloning. If the product appears clean, the PCR reaction can be used directly in the assembly reaction.
- In a PCR tube, combine equimolar amounts of insert fragments in a volume of 7 μl with 3 μl of the linearized pGNW vector. Add 1 μl of 1 U μl^−1^ *USER* enzyme (New England BioLabs, Ipswich, MA, USA). Set up a thermocycler and run a reaction programme as follows: *deoxyuracil excision*, 30 min at 37°C; *annealing 1*, decrement from 28°C to 20°C, −2°C per step, 3 min step duration; and *annealing 2*, ≥ 10 min at 10°C. If a plasmid was used as template for the amplification of one of the fragments that contains the same antibiotic resistance as the employed pGNW vector, add 0.5 μl of *FastDigest Dpn*I (Thermo Fisher Scientific, Waltham, MA, USA) to the reaction mix prior to incubation at 37°C.
- Transform 50 μl of chemically‐competent *E. coli* DH5α λ*pir* cells with 5 μl of the assembly reaction from the previous step. Plate the cells on LB medium agar supplemented with the respective antibiotic for the pGNW vector used. If a circular plasmid was used as template in the PCR for pGNW linearization, and if the reaction was directly employed for the assembly reaction (rather than using a gel‐purified plasmid), spread the transformed *E. coli* DH5α λ*pir* cells on plates containing 5‐bromo‐4‐chloro‐3‐indolyl‐β‐D‐galactopyranoside (Xgal) at 40 μg ml^−1^.

#### Verification of clones

- Use primers Seq‐pGNW_F and Seq‐pGNW_R (Table S1) in a colony PCR (20 μl reaction volume for each PCR) to amplify the HR insert in pGNW of eight individual *E*. *coli* transformants that show green fluorescence under blue‐light exposure (and are white under white light if Xgal was added to the plate).
- Analyse a 3‐ to 5‐μl aliquot of each PCR by agarose gel electrophoresis to verify the correct insert size. Purify the remaining volume of the PCRs with the correct insert size and send the samples for sequencing to verify sequence integrity.
- Inoculate 3–5 ml LB cultures (add the corresponding antibiotic) with six individual clones that were tested for a correct insert size. Incubate the culture at 37°C for 12–18 h in a shaking incubator at 180–250 rpm (depending on the type of incubator). In order to continue with the genome engineering procedure in *Pseudomonas* on the subsequent day (section ‘Genome editing in *Pseudomonas putida*’), launch a pre‐culture of strain KT2440 as well, and incubate with shaking (180–250 rpm) at 30°C.
- Purify plasmid DNA from the *E*. *coli* cultures that tested positive by colony PCR and send the purified plasmid DNA for sequencing in addition to (or instead of) step 2.

#### Cloning of sgRNAs into vector pS448·CsR for counterselection of mutants

To adopt CRISPR‐Cas9 as a counterselection tool, vector pS448**·**CsR was constructed by *USER* assembly from vector pSEVA448 (Table 1) *via* reverse amplification with primers pS448_F and pS448_R (Table S1) and assembled with (i) *cas9*, amplified from plasmid pSEVA421.Cas9tr with primers Cas9_F and Cas9_R and (ii) a *P*_*EM7*_→sgRNA module (synthesized by Integrated DNA Technologies, Leuven, Belgium; and amplified with primers sgRNA_F and sgRNA_R). This module contains a single guide RNA construct fused to a *trans*‐activating CRISPR RNA (*tracr*RNA)‐part and two *Bsa*I recognition sites for the easy insertion of a spacer. The two *Bsa*I recognition sites are placed in inverse orientation immediately upstream of the *tracr*RNA part to enable the creation of incompatible, single‐stranded overhangs during linearization by *Bsa*I. This step allows for the insertion of a double‐stranded DNA (dsDNA) fragment with suitable overhangs. If CRISPR‐Cas9 is to be used as counterselection tool, vector pS448**·**CsR must be equipped with an adequate spacer (Bhaya *et al*., 2011), which targets the original sequence that becomes modified, as disclosed below.

#### Spacer design

For the use of CRISPR‐Cas9 as counterselection tool, a spacer is inserted into vector pS448**·**CsR to target the original sequence that becomes modified after genome engineering. The spacer represents the CRISPR RNA (crRNA)‐part of the sgRNA and is defined by the 20‐nt sequence upstream of a spacer adjacent motif (PAM, 5′‐NGG‐3′) on either of both DNA strands within a target DNA region. Strand specificity is not relevant when CRISPR‐Cas9 is employed for counterselection, and the most important parameter is the sgRNA specificity for its target. To choose a suitable spacer sequence for counterselection, we recommend the use of the online tool *CRISPy‐web* (Blin *et al*., 2016) as indicated below.

- Upload a GenBank file with the complete genomic sequence of your *Pseudomonas* species into CRISPy‐web, available at https://crispy.secondarymetabolites.org/.
- Specify either the gene that is to be deleted/substituted or the range of the MR.
- Pick a suitable 20‐nt spacer candidate sequence from the top entries in the list provided by the CRISPy‐web application.
- Design and order two oligonucleotides that are complementary to each other. The first oligonucleotide represents the 20 nt of the spacer sequence obtained in the previous step. The second oligonucleotide is the reverse complement of the first oligonucleotide. Add a 5′‐AAAC‐3′ overhang to the 5′‐end of the second oligonucleotide and a single C residue to its 3′‐end. Add a 5′‐GCGCG‐3′ overhang to the 5′‐end of the first oligonucleotide. For example, if *aceF* (*PP_0338*) is to be targeted, the sequences would be oligonucleotide 1 (*aceEF*_F), 5′‐**GCGCG** CTC ATT CGC GTA CCT GAC AT **C**‐3′; and oligonucleotide 2 (*aceEF*_R), 5′‐**AAAC** ATG TCA GGT ACG CGA ATG AG **C**‐3′ Table S1 in the Supporting Information; additions are indicated in boldface). For ligation, the oligonucleotides need to be phosphorylated at the 5′‐OH terminus. The oligonucleotides can either be purchased with terminal phosphorylation or phosphorylated *in situ* using T4 polynucleotide kinase (PNK, see below).

#### DNA preparation and construction of derivatives of vector pS448·CsR

- Digest vector pS448**·**CsR with *Bsa*I or *Eco*31I (Thermo Fisher Scientific) according to the manufacturer's recommendations. Use agarose gel electrophoresis and gel purification to isolate the linearized plasmid (9.9 kb) from the non‐restricted fraction.
- Dissolve the two spacer oligonucleotides at 100 μM. Phosphorylate and anneal the oligonucleotides in a thermocycler (Ruiz *et al*., 2006; Jiang *et al*., 2013). This can be performed in a single 10‐μl reaction containing 6 μl of water, 1 μl of each oligonucleotide, 1 μl of T4 ligase buffer and 1 μl of T4 PNK (New England BioLabs). Use the following temperature protocol: 30 min at 37°C, 4 min at 95°C, followed by 70 cycles consisting of 12 s each, starting at 95°C and decreasing the temperature by 1°C in each cycle.
- Dilute the annealed and phosphorylated oligonucleotides 1:200 with water, i.e. to a final concentration of dsDNA of 50 nM.
- Ligate the dsDNA encoding the sgRNA‐spacer into the linearized pS448**·**CsR vector in a 10‐μl reaction containing 3 μl of diluted insert from the previous step, 10–30 ng of *Bsa*I‐digested pS448**·**CsR vector, 1 μl of T4 ligase buffer, 1 μl of T4 DNA ligase (New England BioLabs) and water, if needed, to reach the final volume.
- Ligate 30 min at room temperature and transform a 50‐μl aliquot of chemically competent *E. coli* DH5α cells with 5 μl of the ligation mixture. Plate on LB medium agar supplemented with streptomycin.
- Purify plasmid DNA from three individual *E. coli* transformants and verify the sequence integrity by sequencing with primer SEVA‐T0_F (Table S1).

#### Test efficiency of the sgRNA for counterselection

This step is optional and is meant to provide an estimation of the efficiency of the sgRNA in targeting the locus targeted during the procedure (thus providing an estimate of the success rate of the whole counterselection procedure).

- Inoculate 10 ml of LB medium with *P*. *putida* KT2440 and grow the cells overnight at 30°C with agitation.
- Wash the cells four times with 1 ml of 300 mM sucrose (filter‐sterilized) and finally resuspend in 400 μl of 300 mM sucrose.
- Individually electroporate 100 ng of the empty pS448**·**CsR vector and 100 ng of plasmid pS448**·**CsR carrying the sgRNA of choice into 100‐μl cell suspension aliquots with a voltage of 2.5 kV, 25 μF capacitance and 200 Ω resistance (e.g. in a Gene Pulser Xcell™ Electroporation System; Bio‐Rad Laboratories, Hercules, CA, USA).
- Let the cells recover for 2 h at 30°C and plate them onto the LB medium agar supplemented with streptomycin (100 μg ml^−1^). Incubate the plates for overnight at 30°C.
- The plate with cells harbouring vector pS448**·**CsR with a functional sgRNA should have considerably less colonies (if any at all). We typically see a number of CRISPR‐Cas9 escapers in the low double‐digit range (i.e. 10–20 colonies) while *Pseudomonas* transformed with an empty pS448**·**CsR vector will form several hundred colonies within 16 h.

### Genome editing in *Pseudomonas putida*

**Figure 3:**
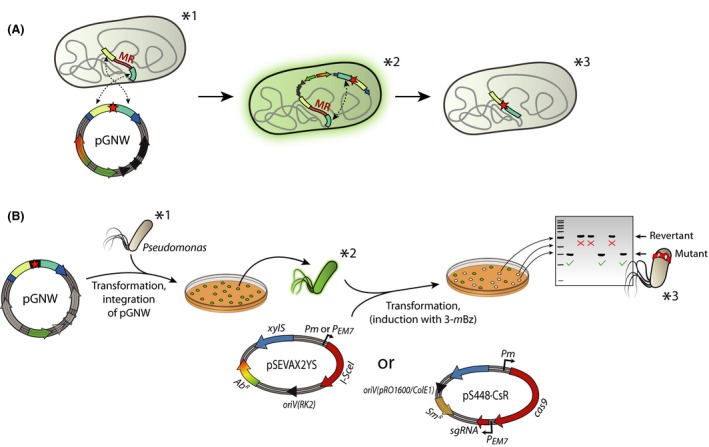
Workflow for targeted genomic manipulations in *Pseudomonas*. *Pseudomonas* cells are transformed with pGNW *via* electroporation or tri‐parental mating. One to five individual green fluorescent colonies are combined and enriched in liquid LB medium cultures and transformed either with plasmid pSEVAX2YS or with a derivative of vector pS448**·**CsR carrying an appropriate sgRNA. Note that the antibiotic resistance could be different depending on the vector chosen for the deletion. Expression of the gene encoding the I‐*Sce*I meganuclease mediates the excision of the pGNW sequence from the chromosome, leading to non‐fluorescent colonies under blue‐light exposure. These clones are tested *via* colony PCR and sequencing for a revertant (i.e. wild‐type) or mutant genotype. Asterisks indicate the relevant genotypes as well as the molecular events happening in the *Pseudomonas* chromosome (A) during the corresponding steps of the genome engineering protocol (B).

In order to introduce mutations into the *Pseudomonas* genome, the suicide plasmid pGNW (constructed in the previous section) is integrated into the chromosome at the target location employing the native homologous recombination mechanism (Fig. 3). After selection of positive co‐integration events, the meganuclease I‐*Sce*I or the CRISPR‐Cas9 system, respectively, is delivered into the cells on a replicative plasmid. The expression of the DNA modifying enzymes – and thus restriction of pGNW within the chromosome – enforces a second homologous recombination that can yield either the mutant genotype or a revertant genotype (i.e. wild‐type sequence).

#### Integration of pGNW derivatives into the chromosome

The suicide vector pGNW carrying the corresponding HRs to the MR has to be delivered into the cells to enable its chromosomal integration *via* homologous recombination. This step can be achieved either by electroporation or by tri‐parental mating. The latter procedure is recommended if multiple manipulations are conducted within the same strain. Thereby, the plasmid bearing I‐*Sce*I (e.g. pSEVAX28S, where X indicates any antibiotic resistance; see Table 1) can be maintained within the cells by addition of the respective antibiotic during all cultivation steps. A leaky expression of the gene encoding I‐*Sce*I, however, interferes with the integration of pGNW – significantly decreasing the transformation efficiency. This issue can be countered by a much more efficient plasmid delivery achieved by tri‐parental mating.

### Plasmid delivery by electroporation

- Grow an overnight culture of *P*. *putida* KT2440 in 5–10 ml of LB medium. The *Pseudomonas* culture can be started at the same time that the overnight cultures of *E*. *coli* DH5α λ*pir* with pGNW (Section ‘Verification of clones’, step 3).
- For the electroporation of cells, we adopted the procedure by Choi *et al*. (2006). In brief, the cell suspension is distributed into four microcentrifuge tubes and pellet the cells in a microcentrifuge at 11 000 *g* for 1 min. Wash the cells twice with 1 ml of 300 mM sucrose for each pellet and finally resuspend and combine them in a total of 100 μl of 300 mM sucrose in a single tube. Add 500 ng of pGNW vector to the suspension and transfer the mixture into an electroporation cuvette with 2 mm gap width. Apply an electric field pulse (2.5 kV voltage, 25 μF capacitance, and 200 Ω resistance) and quickly add 1 ml of LB medium. All steps of the electroporation protocol can be performed at room temperature.
- Transfer the cell suspension into a sterile test or centrifuge tube (5–50 ml) and recover the cells with shaking at 30°C for 2 h.
- Harvest the cells by centrifugation (11 000 *g*; 1 min) at room temperature, and plate the whole suspension onto LB medium agar supplemented with the respective antibiotic for the pGNW vector used (either 50 μg ml^−1^ Km, 10 μg ml^−1^ Gm or 100 μg ml^−1^ Sm). Incubate the cells overnight at 30°C.
- Inspect the plate on a blue‐light transilluminator. Transformants should show green fluorescence (usually all colonies).

### Plasmid delivery by tri‐parental mating

- Grow overnight cultures of *Pseudomonas*,* E. coli* DH5α λ*pir*/pGNW and *E. coli* HB101/pRK600 in at least 1 ml of LB medium each. Incubate at 30°C for *Pseudomonas* or 37°C for *E. coli* respectively.
- Dry an LB medium agar plate without added antibiotics in a clean bench for 1 h.
- Combine 100 μl of each pre‐culture into a reaction tube and pellet the cells in a microcentrifuge at 10 000 *g* for 1 min at room temperature. Wash the cells with 1 ml of fresh LB medium and finally resuspend them in 20–30 μl of LB medium. Pipette the whole suspension onto the pre‐dried LB medium agar plate. In order for bacterial conjugation to work, the cells have to be brought into close proximity within a non‐planktonic state. A dried agar plate will absorb a small volume of media, allowing the bacterial cells to form a biofilm‐like structure on the agar surface.
- Incubate the cells at 30°C for 3–5 h.
- Use an inoculation loop to take the biomass up from the agar surface and resuspend it in 1 ml of LB medium. Spread 100 μl of this suspension directly onto a cetrimide agar plate supplemented with the antibiotic to select for the pGNW vector used, as well as 50 μg ml^−1^ ampicillin, and the antibiotic for the I‐*Sce*I plasmid (if already delivered at this step, see below). Concentrate the remaining cells to about 100 μl by centrifugation, and resuspend and spread them onto a second plate. The chosen concentration of Amp was found to not affect the growth of *P*. *putida* KT2440 that is naturally resistant to *β*‐lactam antibiotics while completely suppressing the growth of most *E*. *coli* strains, which may show some residual growth even in the presence of cetrimide.
- Incubate the plates at 30°C for 16–24 h until clear colonies have formed that show green fluorescence under blue‐light exposure.

### Introduction of endonucleases

- Combine 1–5 individual colonies of *Pseudomonas* with a co‐integrated pGNW vector into 5–10 ml of LB medium in a test or centrifuge tube (5–50 ml) and incubate with shaking at 30°C for at least 5 h (see next step). Using several individual colonies increases the chances of having cells with both the two possible genetic configurations after pGNW co‐integration (Fig. 5A), and reduces the impact of unspecific insertion of the suicide plasmid at untargeted chromosomal sites. Furthermore, the more biomass is used to inoculate the LB medium culture, the quicker the cell density required for the next step is reached so that the protocol can be continued on the same day. If the cells still contain an inducible I‐*Sce*I―plasmid (i.e. pSEVAX28S) from prior mutagenesis procedures, continue directly with Step 3 below.
- After the cells have reached an optical density at 600 nm (OD_600 nm_) of at least 0.3, use 20–100 ng of an I‐*Sce*I plasmid (i.e. pSEVAX2YS, where the expression of I‐*Sce*I could be either constitutive or inducible; see Table 1) or 100–200 ng of plasmid pS448**·**CsR to transform *Pseudomonas* co‐integrants by electroporation as described in the previous section (Section ‘Plasmid delivery by electroporation’). Transfer the cell suspension into a sterile test or centrifuge tube and recover the cells shaking at 30°C for 1 h. If a plasmid containing the XylS/*Pm* expression system (i.e. pSEVAX28S or pS448**·**CsR) is used, add 3‐*m*Bz to a final concentration of 3 mM to the LB medium used for cell recovery.
- Plate 70 μl of cell suspension on LB medium agar supplemented with the respective antibiotic for pSEVAX2YS (i.e. 500 μg ml^−1^ Amp, 50 μg ml^−1^ Km, 10 μg ml^−1^ Gm or 100 μg ml^−1^ Sm), or Sm for pS448**·**CsR selection. Incubate the cells overnight at 30°C. If a plasmid containing the XylS/*Pm* expression system (i.e. pSEVAX28S or pS448**·**CsR) is used, add 3‐*m*Bz to a final concentration of 3 mM to the LB medium plates to ensure proper induction of the system.
- Inspect the plate on a blue‐light table. *Pseudomonas* clones that have lost vector pGNW through a second homologous recombination event will show no green fluorescence.
- Test ≥ 8 individual non‐fluorescent colonies *via* colony PCR using the *g*‐check primers adequate for their genotype. If CRISPR‐Cas9 counterselection was employed, all non‐fluorescent clones are expected to harbour the mutation. The fraction of cells that have maintained fluorescence after counterselection is expected to be significantly higher when using CRISPR‐Cas9 as compared to I‐*Sce*I, due to a higher probability of escaping restriction by mutating the sgRNA recognition sequence.

### Plasmid curing

**Figure 4:**
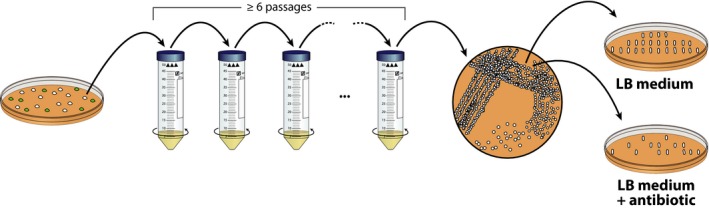
Plasmid curing after successful genome engineering of *Pseudomonas*. The biomass from a single mutant *Pseudomonas* colony is used to inoculate 5**–**10 ml of LB medium and the suspension is incubated at 30°C with shaking. Two to three times per day, a small volume of this culture is transferred into fresh LB medium. After 2–3 days (i.e. at least six passages), the cells are isolated on LB medium agar plates and tested for the loss of plasmids based on their antibiotic‐resistance profile.

The last step in the procedure, once the mutation has been confirmed, is to cure the mutant cells from the plasmid(s) used during genome editing (Fig. 4).

- Use a single colony of mutant *Pseudomonas* to inoculate 3–10 ml of LB medium in a test or centrifuge tube.
- Culture the cells shaking at 30°C. Every 4–12 h, transfer 1 μl of the culture into 3–10 ml of fresh LB medium. Continue the culturing process for 2–3 days, and pass the cells into fresh medium even if you cannot see the formation of biomass in the tubes. This procedure ensures that the cells are kept in a state of maximum division rate. If your mutant cells have severe growth deficiencies due to the deletion of important genes or the introduction of DNA that could impair growth, the curing of a plasmid can require continuous cultivation for an extended period.
- Dilution‐streak the culture on an LB medium agar plate without added antibiotics.
- Test individual colonies for the loss of the plasmid by re‐streaking on LB medium agar with the respective antibiotic.

## Application examples

### Deletion of *nicX* in *Pseudomonas putida* KT2440 mediated by I‐*Sce*I activity

**Figure 5:**
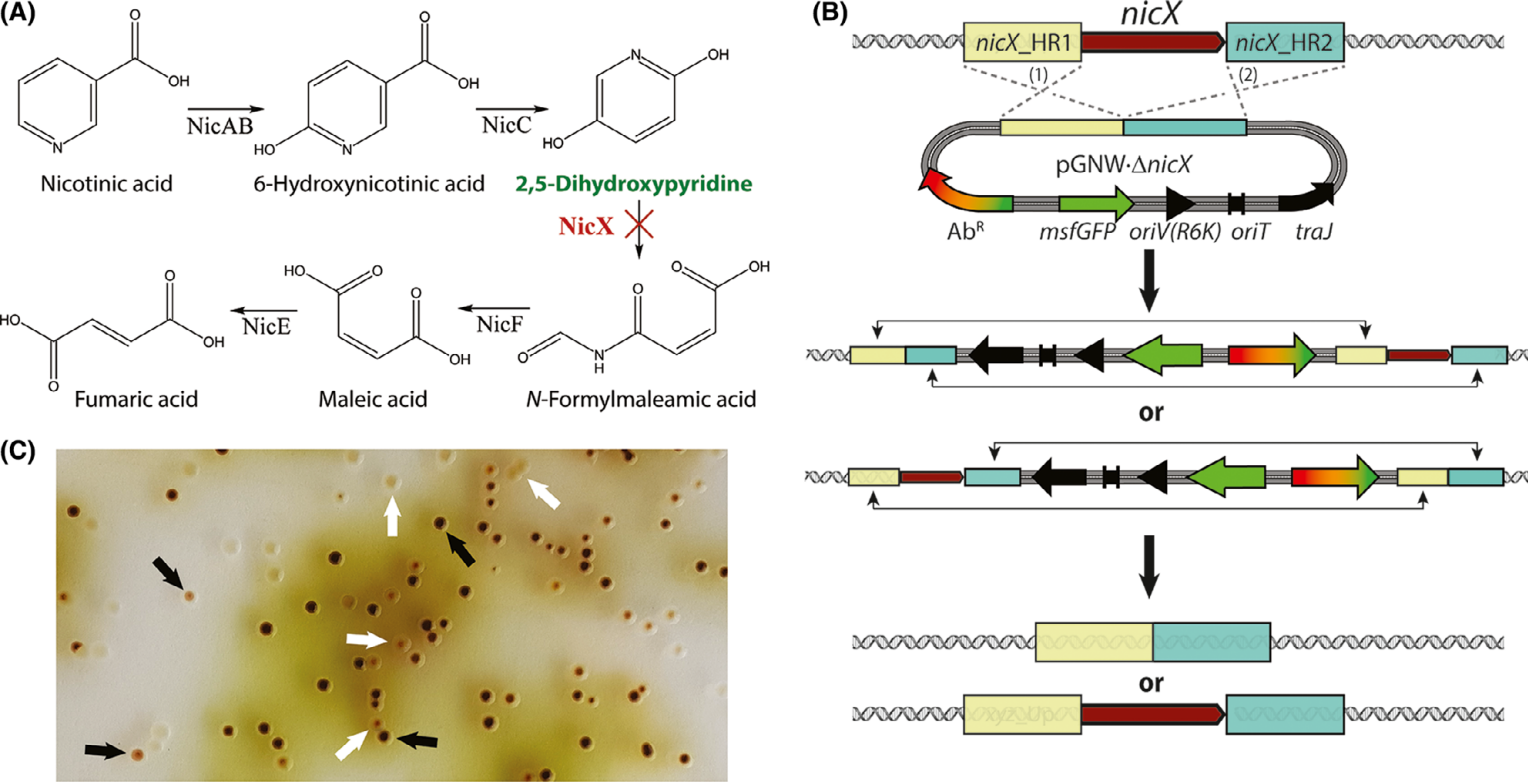
Deletion of *nicX* in *Pseudomonas putida* KT2440. A. Proposed degradation pathway of nicotinic acid in *P*. *putida* based on the information available in the literature (Jiménez *et al*., 2008). The key metabolic intermediate accumulating upon elimination of *nicX* (encoding 2,5‐dihydroxypyridine‐5,6‐dioxygenase) is highlighted in green. The targeted, in‐frame deletion of *nicX* is indicated by a red cross. B. Schematic representation of the molecular mechanism for the integration of the suicide plasmid pGNW **·**Δ*nicX* in the chromosome of strain KT2440 as well as the second recombination leading to either a revertant or a mutant genotype. Note that the antibiotic resistance could be different depending on the vector chosen for the deletion. C. Representative picture of a section of an LB medium agar plate (containing 10 μg ml^−1^ Gm and 5 mM nicotinic acid) seeded with an isolate of strain KT2440 that was previously co‐integrated with the suicide plasmid pGNW2·Δ*nicX* and transformed with the helper plasmid pSEVA6213S. The accumulation of 2,5‐dihydroxypyridine (green‐to‐brown pigmented colonies) can be easily detected by visual inspection of the plates. The picture was taken after incubation for 16 h at 30°C followed by 24 h at 4°C. The black arrows indicate colonies formed by *P*. *putida* Δ*nicX* cells; the white arrows identify colonies displaying a revertant (i.e. wild‐type) genotype.

*Pseudomonas putida* KT2440 is able to grow on nicotinic acid as a sole carbon source (Belda *et al*., 2016). The degradation pathway, encoded in the *nic* gene cluster, involves the hydroxylation of nicotinate to 6‐hydroxynicotinate, its further reduction to 2,5‐dihydroxypyridine (Fig. 5A), and the deoxygenation to *N*‐formylmaleamic acid, which can be further converted into fumarate (Jiménez *et al*., 2008). An interruption of the metabolic route at the level of 2,5‐dihydroxypyridine *via* the deletion of *nicX* (encoding a 2,5‐dihydroxypyridine 5,6‐dioxygenase) leads to the accumulation of a dark green coloured compound with green fluorescence within and outside of the cells (Jiménez *et al*., 2015), which forms brown polymers upon autoxidation. A deletion of *nicX* in strain KT2440 was thus chosen to optimize the genome engineering protocol since it allows for the direct identification of mutant clones after addition of nicotinic acid to the culture medium.

The primer pairs *nicX*_HR1_F/*nicX*_HR1_R and *nicX*_HR2_F/*nicX*_HR2_R (Table S1) were used to amplify the two homology regions that flank *nicX,* and the products were assembled and cloned into vectors pGNW2, pGNW4 and pGNW6 *via USER* assembly, yielding pGNW2·Δ*nicX*, pGNW4·Δ*nicX* and pGNW6·Δ*nicX* respectively (Fig. 5B and Table 1). After their purification from *E. coli* DH5α λ*pir*, the insertional plasmids were integrated into the chromosome of strain KT2440 by electroporation. Then, the successful co‐integrant clones were transformed with either plasmid pSEVA6213S (in clones where either vector pGNW2·Δ*nicX* or pGNW4·Δ*nicX* was used for the integration) or plasmid pSEVA1213S (in clones where vector pGNW6·Δ*nicX* was used for the integration step). Cells were plated on LB medium agar containing Gm or Amp respectively. Figure 5C shows a photograph of an LB medium agar plate supplemented with Gm and 5 mM nicotinic acid, taken 1 day after transforming *P. putida* KT2440/pGNW2·Δ*nicX* with plasmid pSEVA6213S. Thirty randomly picked colonies from the plates obtained after each deletion procedure were tested for their genotype *via* colony PCR using primers *nicX*_*g*‐check_F/*nicX*‐*g*‐check_R (Table S1). The different colorations of mutant and revertant colonies allowed for their direct identification. With all three plasmid combinations, the fraction of mutant colonies was always close to 50% (53%, 46% and 48% respectively).

### Deletion of *aceEF* in *Pseudomonas putida* KT2440 using CRISPR‐Cas9 counterselection

As an obligate aerobic bacterium, *P. putida* strongly relies on an active tricarboxylic acid cycle for the generation of reducing equivalents that are used, *inter alia*, to generate ATP through oxidative phosphorylation (Nikel *et al*., 2014). The pyruvate dehydrogenase complex (PDHc), which catalyses the entry reaction to the tricarboxylic acid cycle, plays a central role in the central carbon metabolism of *Pseudomonas* (Sudarsan *et al*., 2014; Nikel *et al*., 2015). The PDHc is a multimeric system composed of three different proteins, AceE, AceF and Lpd (Reed *et al*., 1975; Angelides *et al*., 1979). In *P. putida* KT2440, the genes encoding AceE (*PP_0339*) and AceF (*PP_0338*) form an operon (Belda *et al*., 2016). Considering its central role in sugar catabolism, genes encoding PDHc are considered a difficult knock‐out to achieve if relying on an untargeted selection strategy, and the resulting mutant would become bradytroph for C2 units as the reaction converting pyruvate into acetyl‐coenzyme A is blocked. Because of this reason, CRISPR‐Cas9 was adopted for counterselection of the correct mutants in this study. The homology regions to delete *aceE* and *aceF* were amplified from chromosomal DNA of *P. putida* KT2440 using the primer pairs *aceEF*_HR1_F/*aceEF*_HR1_R and *aceEF*_HR2_F/*aceEF*_HR2_R (Table S1) and cloned into vector pGNW2. *Pseudomonas* co‐integrants were then transformed with 200 ng of plasmid pS448**·**CsR_*aceF* (Table 1) and selected on LB medium agar supplemented with Sm, 3‐*m*Bz and 5 mM acetate to enhance the growth of bradytrophic mutant cells. After 2 days, 10 out of 39 colonies of normal size and a majority (29 out of 39) of very small colonies could be identified on the plate (Fig. S2). Inspection under blue‐light exposure revealed that all larger colonies had kept green fluorescence and thus had escaped CRISPR‐Cas9‐mediated restriction. All smaller colonies were tested by colony PCR with primers *aceEF*_g‐check_F/*aceEF*_g‐check_R (Table S1) and were found to have *aceEF* deleted. In a separate experiment, co‐integrant cells were transformed with both pSEVA628S and pS448**·**CsR_*aceF* (500 ng of each plasmid). Selection on LB medium agar plates containing Sm, 3‐*m*Bz, and acetate (as a direct source of acetyl‐coenzyme A) led to the formation of only three colonies, all of which had *aceEF* deleted.

### Integration of fluorescent protein‐encoding genes into a landing pad in *Pseudomonas putida* KT2440

To evaluate the suitability of the system for the insertion of DNA at a defined position, a landing pad was chosen within the intergenic region between *PP_0013* (*gyrB*) and *PP_5421*, close to the chromosomal origin of replication of *P.** **putida* KT2440 (Nelson *et al*., 2002; Reynolds and Gill, 2015; Belda *et al*., 2016). First, plasmid pGNW2·LPR was assembled from four fragments in a *USER* assembly reaction. To this end, vector pGNW2 was amplified with pGNW‐*USER*_F and pGNW‐*USER*_R. The two HRs framing the landing pad were amplified from genomic DNA with the primer pairs LP_HR1_F/LP_HR1_R and LP_HR2_F/LP_HR2_R (Table S1). A fourth fragment containing the gene encoding the monomeric red fluorescent protein mRFP1 (Campbell *et al*., 2002) and the *rrnB*‐T1 terminator element was amplified with RFP4LP_F and RFP4LP_R (Table S1) from vector pS2313·R. The primers RFP4LP_F and LP_HR1_R furthermore contained extended overhangs to introduce the constitutive *P*_*EM7*_ promoter upstream of the *mRFP1* coding sequence. Since this promoter was found to give a too low expression (data not shown), its sequence was exchanged with the strong *P*_*14g*_ promoter (Zobel *et al*., 2015) and the translational coupler *BCD2* (Mutalik *et al*., 2013) by amplifying pGNW2·LP::*P*_*EM7*_‐mRFP with oligonucleotides *P14 g*‐*BCD2*_F/*P14 g*‐*BCD2*_R and a *BCD2*‐fragment from a gBlock containing this regulatory element (purchased from Integrated DNA Technologies) with *BCD2*‐*P14g*_F/ *BCD2*‐*P14g*_R. The resulting plasmid was termed pGNW2·LPR (Table 1).

To exchange *mRFP1* in the landing pad with different fluorescent proteins and to transfer the landing pad to vectors pGNW4 or pGNW6, two‐ or four‐fragment *USER* assembly reactions were performed with interchangeable modules. These modules were amplified and assembled as indicated in Table S1. The *msfGFP* gene was amplified from vector pGNW4. The genes encoding the other fluorescent proteins were amplified from the plasmids pS2313·B, pS2313·O and pS2313·T that had been constructed as follows: The sequences of the genes *mBFP2*,* mOrange2* and *mTurquoise2* were extracted from their original publications (Table 1), cured from protein tags and restriction sites to make them SEVA‐compatible and ordered as custom genes (Integrated DNA Technologies). The synthetic DNA fragments were then cloned into the expression vector pSEVA2313 *via USER* assembly using the primer pairs pS2313_F/pS2313_R (for pSEVA2313), pS_BFP2_F/pS_BFP2_R (for *mBFP2*) and pS_Ora_Tq _F/pS_Ora_Tq _F (for *mOrange2* and *mTurquoise2*; Table S1), yielding plasmids pS2313·B, pS2313·O and pS2313·T respectively.

**Figure 6:**
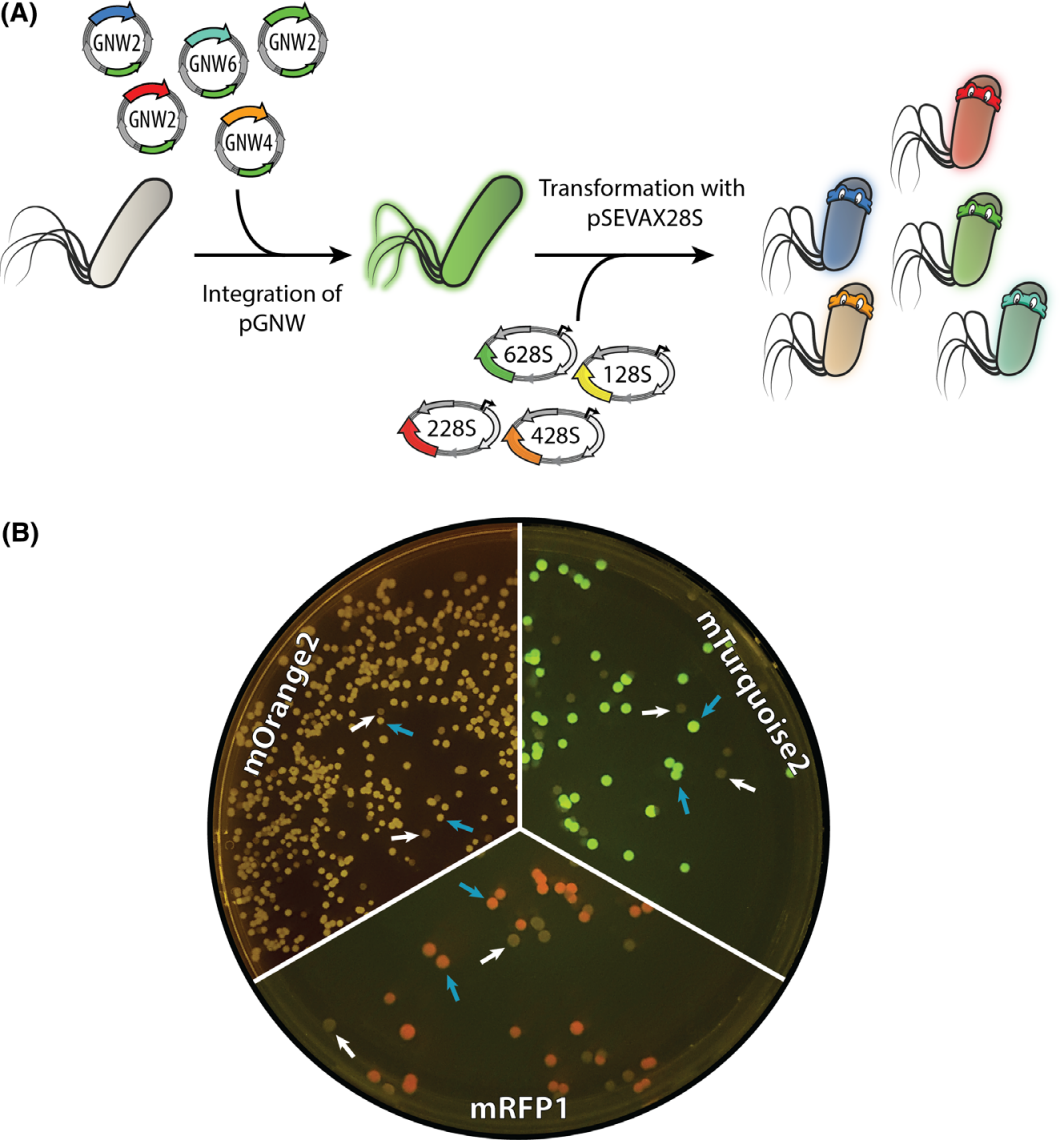
Integration of different fluorescent proteins into a landing pad in the chromosome of *Pseudomonas putida* KT2440. A. Genes encoding the fluorescent proteins mRFP1, mOrange2, mTurquoise2, msfGFP, and mBFP2 [placed under transcriptional control of a *P*_*14g*_(*BCD2*) regulatory element], were integrated into a chosen landing site in the chromosome of *P.** **putida* KT2440 using the insertional vectors pGNW2, pGNW4, or pGNW6. After selection of co‐integrants, the second homologous recombination was mediated by the inducible expression of the gene encoding the I‐*Sce*I meganuclease from pSEVAX28S vectors. B. Images of the plates were taken after incubation for 16 h at 30°C followed by 7 days at 4°C to allow for proper fluorescent protein maturation. *mBFP2*‐integrated colonies showed no visible fluorescence under blue‐light exposure (not shown). The blue arrows indicate colonies of mutated strains displaying different integrated fluorescent proteins; white arrows identify colonies of cells displaying a revertant genotype (i.e. wild‐type) without fluorescence.

The resulting plasmids pGNW2·LPR, pGNW2·LPG, pGNW2·LPB, pGNW4·LPO and pGNW6·LPT (Table 1) were individually integrated into the chromosome of *P. putida* KT2440 as indicated above. The backbones were removed from the co‐integrants by the delivery and induction of plasmid pSEVA128S (for pGNW2·LPR), pSEVA628S (for pGNW2·LPG and pGNW2·LPB), pSEVA228S (for pGNW4·LPO) and pSEVA428S (for pGNW6·LPT) respectively (Fig. 6A). On the following day, the plates with the resulting strains (*P*. *putida* KT·LPR, KT·LPG, KT·LPB and KT·LPO; Table 2) were placed at 4°C to let the fluorescent proteins mature, since none of them showed any visible coloration under blue‐light exposure. After 7 days, photographs of the plates were taken on a blue‐light transilluminator (Fig. 6B). *Pseudomonas* colonies with integrated *mBFP2* (strain KT·LPB) showed not visible fluorescence even after further prolonged incubation (data not shown). To determine the kinetics of the fluorescent proteins in *P.** **putida* KT2440, at least three biological replicates of the mutant strains (verified by colony PCR with primers Seq‐LP_F and Seq‐LP_R, Table S1) and the wild‐type strain were grown at 30°C in 96‐well plates (Greiner CELLSTAR™; Sigma‐Aldrich, St. Louis, MO, USA; polystyrene, round bottom) with 200 μl of de Bont minimal medium (Hartmans *et al*., 1989) per well, supplemented with 30 mM citrate, and covered with a sealing membrane (Diversified Biotech Breathe‐Easy™; VWR, Radnor, PA, USA). The kinetics of bacterial growth and fluorescence were acquired by measuring the OD_630_ as well as the excitation/emission values of mRFP1 at 582 nm/609 nm, mBFP2 at 385 nm/450 nm, mOrange2 at 541 nm/567 nm and mTurquoise2 at 451 nm/477 nm (Fig. S1). Although under the transcriptional control of the same regulatory elements, the five fluorescent proteins differed in both the intensity of their signal as well as their expression pattern. The fluorescence intensity of only msfGFP increased steadily for the whole cultivation time of 50 h; the signal of the remaining four reporter proteins reached stagnation after 15 h (mOrange2), 8 h (mRFP1 and mBFP2) and 25 h (mTurquoise2). Since all cultured strains continue to grow until the end of the experiment, the biomass‐specific fluorescence decreased continuously (Fig. S1).

**Table 2:** Bacterial strains used in this study

Strain	Relevant characteristics^a^	Reference or source
*Escherichia coli*		
DH5α	Cloning host; F^–^ λ^–^ *endA1 glnX44*(AS) *thiE1 recA1 relA1 spoT1 gyrA96*(Nal^R^) *rfbC1 deoR nupG* Φ80(*lacZ*Δ*M15*) Δ(*argF*‐*lac*)*U169 hsdR17*(*r*_*K*_^−^ *m*_*K*_^*+*^)	Hanahan and Meselson (1983)
DH5α λ*pir*	Cloning host; same as DH5α but λ*pir* lysogen	Platt *et al*. (2000)
HB101	Helper strain used for tri‐parental mating; F^–^ *thi‐1 hsdS20*(r_B_^−^ m_B_^−^) *supE44 recA13 ara‐14 leuB6 proA2 lacY1 galK2 rpsL20*(Sm^R^) *xyl‐5 mtl‐1*	Boyer and Roulland‐Dussoix (1969)
*Pseudomonas putida*		
KT2440	Wild‐type strain, derived from *P*. *putida* mt‐2 (Worsey and Williams, 1975) cured of the TOL plasmid pWW0	Bagdasarian *et al*. (1981)
KT2440 Δ*aceEF*	Same as KT2440, but with an in‐frame deletion of the *aceEF* genes (*PP_0038*‐*PP_0339*)	This work
KT2440 Δ*nicX*	Same as KT2440, but with an in‐frame deletion of the *nicX* gene (*PP_0395*)	This work
KT·LPR	Same as KT2440, but carrying a *P*_*14g*_(*BCD2*)→*mRFP1* element integrated between *PP_0013* (*gyrB*) and *PP_5421*	This work
KT·LPG	Same as KT2440, but carrying a *P*_*14g*_(*BCD2*)→*msfGFP* element integrated between *PP_0013* (*gyrB*) and *PP_5421*	This work
KT·LPB	Same as KT2440, but carrying a *P*_*14g*_(*BCD2*)→*mBFP2* element integrated between *PP_0013* (*gyrB*) and *PP_5421*	This work
KT·LPO	Same as KT2440, but carrying a *P*_*14g*_(*BCD2*)→*mOrange2* element integrated between *PP_0013* (*gyrB*) and *PP_5421*	This work
KT·LPT	Same as KT2440, but carrying a *P*_*14g*_(*BCD2*)→*mTurquoise2* element integrated between *PP_0013* (*gyrB*) and *PP_5421*	This work

**a.** Antibiotic markers: *Nal*, nalidixic acid; and *Sm*, streptomycin.

## Discussion

The present genome engineering protocol reduces the hands‐on work of the original procedure by Martínez‐García and de Lorenzo (2011). We further streamlined the protocol with (i) the adoption of the *USER* assembly method that enables a standardized workflow (Cavaleiro *et al*., 2015), (ii) a reporter function (i.e. fluorescence) for the donor plasmid that allows for its direct visualization within host cells (Calero *et al*., 2016), (iii) additional antibiotic resistances to broaden the host spectrum and (iv) an efficient CRISPR‐Cas9 counterselection, particularly relevant for the construction of difficult knock‐outs. The protocol described herein yields reliable results for targeted mutagenesis in *Pseudomonas* species within a standard workweek.

We have illustrated the flexibility of the system by combining different molecular elements of the toolbox to delete genomic regions in *P. putida* KT2440 and demonstrated the functionality of four new fluorescent proteins (mRFP1, mOrange2, mTurquoise2 and, to some extent, mBFP2) in this host *via* their targeted integration into a landing site in the chromosome. While we optimized the critical steps of the protocol that yield the desired mutation, the subsequent curing of replicating plasmids still adds up time required to generate the final, plasmid‐free strain. One possible solution could be to implement conditional origins of replication in the current plasmid system, e.g. temperature‐sensitive derivatives of *oriV(RK2)* (Valla *et al*., 1991) that has been shown to function in *P. putida* (Choi *et al*., 2018). This will be of particularly value for the curing of plasmid DNA from cells that are severely impaired in growth and are thus less prone to lose plasmids during proliferation. A reliable plasmid curing after each cycle of mutagenesis could also solve issues encountered with the non‐induced, basal expression of the XylS/*Pm* expression system that can be observed particularly with increased copy numbers of the *xylS* gene (Gawin *et al*., 2017) or would allow to rely exclusively on the constitutive system. Furthermore, if combined with the first pre‐culturing step of *Pseudomonas* in this protocol, plasmid curing would not affect its time requirements. While the synthetic biology toolbox for *Pseudomonas* is subjected to continuous improvement, the protocol discussed herein represents the fastest extant procedure for genome editing of *P. putida* and related species.

## Conflict of interest

None declared.

## Supporting information


**Table S1.** Oligonucleotides used in this work.
**Table S2.** Assembly of pGNW plasmids with genes encoding fluorescent proteins within a chromosomal landing pad.
**Fig. S1.** Kinetics of the accumulation of selected fluorescent proteins measured in P. putida KT2440.
**Fig. S2.** Deletion of aceEF in P. putida KT2440 using a synthetic CRISPR‐Cas9 device for counterselection.
