# Supplementary material for: Accelerated genome engineering of Pseudomonas putida by I‐SceI―mediated recombination and CRISPR‐Cas9 counterselection
Source: Microb Biotechnol. 2019 Mar 12;13(1):233–49. doi: 10.1111/1751-7915.13396 (PMC6922521; doi:10.1111/1751-7915.13396)
Supplement: Supplementary file 1 — Table S1. Oligonucleotides used in this work. Table S2. Assembly of pGNW plasmids with genes encoding fluorescent proteins within a chromosomal landing pad. Fig. S1. Kinetics of the accumulation of selected fluorescent proteins measured in P. putida KT2440. Fig. S2. Deletion of aceEF in P. putida KT2440 using a synthetic CRISPR‐Cas9 device for counterselection. [file MBT2-13-233-s001.pdf]

Supporting Information

Accelerated genome engineering of *Pseudomonas putida* by I-*Sce*I—mediated recombination and CRISPR-Cas9 counterselection

by

Nicolas T. Wirth, Ekaterina Kozaeva, and Pablo I. Nikel

Table S1 · Oligonucleotides used in this work.

Name	**Sequence** (5' → 3')	T_m (°C)
pGNW-*USER*_F	AGTCGACCUGCAGGCATGCAAGCTTCT	71.8
pGNW- *USER* _R	AGGATCUAGAGGATCCCCGGGTACCG	66.1
Seq-pGNW_F	TGTAAAACGACGGCCAGT	54.2
Seq-pGNW_R	ATGACCATGATTACGCCGG	55.2
SEVA-*T0*_F	GAACGCTCGGTTGCCGCC	64.4
*nicX*_HR1_F	AGATCCUCATGCGGCGGAAGATTTTC	59.1
nicX _HR1_R	ACCCCTACAUCGGGGTTCTCCTGGG	62.7
nicX _HR2_F	ATGTAGGGGUTATACTGGCCGG	59.9
nicX _HR2_R	AGGTCGACUGCAGCAGGTACAAAATGGC	59.1
nicX _g-check_F	GCCTGACGATTTAGAGCG	53.7
nicX _g-check_R	AAGAAGCAGGCCAGAGATG	54.9
LP_HR1_F	AGATCCUTCATCGAAAGTAACGCGC	58.9
LP_HR1_R	ATGCCGAUATACTATGCCGATGATTAATTGTCAACAGAT CAGGTCTGAAGCTCTCG	58.9
LP_HR2_F	ACCAAAUTGCTCCAGCGATCAC	59.1
LP_HR2_R	AGGTCGACUGAACACGTTCCTCACTGGG	59.6
pS448_F	AGTCGGUGCAATTCGAGCTCGGTACCCG	57.4
pS448_R	ATATGTUTTTCCTCCTAACCGCGGC	58.3
Cas9_F	AACATAUGGATAAGAAATACTCAATAGGCT	52.2
Cas9_R	AGATCAGUCACCTCCTAGCTGACTCA	55.1
sgRNA_F	ACTGATCUAGAGTCGACCTGCAGGCA	57.7
sgRNA_R	ACCGACUCGGTGCCACTTTTTCAAGTTG	57.3
pS2313_F	ACTAGTCTUGGACTCCTGTTGATAGAT	56.0
pS2313_R	ATGTTTUTCCTCCTAAGCTTGCATG	55.8
pS_BFP2_F	ACATATGGUGTCTAAGGGCGAAGAGCTGAT	58.3
pS_BFP2_R	AGTTTAAUTAAGTTTGTGCCCCAGTTTGCT	59.0
pS_Ora_Tq _F	AAAACAUATGGTGAGCAAGGGCGA	57.8
pS_Ora_Tq_R	AAGACTAGUTTACTTGTACAGCTCGTCCAT	58.9
RFP4LP_F	ATCGGCAUAGTATAATACGACAAGGTGAGGAACTAAACC ACTGAGCACTACTAGAGAAAG	55.6
RFP4LP_R	ATTTGGUAGAGAGCGTTCACCGAC	59.5
*P14g-BCD2*_F	AATGGCTUCCTCCGAAGACG	61.2
*P14g-BCD2*_R	AGAGCCUTGTCAATGGGCGATCAGGTCTGAAGCTCTCG	58.9
*BCD2-P14g*_F	AGGCTCUCGCGGCCAGGTATAATTGCACGAGGGCCCAA GTTCACTTAAAAAG	60.9
*BCD2-P14g*_R	AAGCCATUAGAAAACCTCCTTAGCATGA	58.1

*BCD2*_R	AGAAAACCUCCTTAGCATGATTAAG	58.4
pGNW_LP_F	AAGTAAUAACGCTGATAGTGCTAGTG	57.8
BFP2_F	AGGTTTTCUAATGGTGTCTAAGGGCGAAGAG	61.1
BFP2_R	ATTACTUAATTAAGTTTGTGCCCCAGTTTGC	62.3
GFP_F	AGGTTTTCUAATGCGTAAAGGTGAAGAACTGTTC	60.9
GFP_R	ATTACTUATTTGTAGAGTTCATCCATGCCG	64.0
Ora_Tq _F	AGGTTTTCUAATGGTGAGCAAGGGCGAG	61.7
Ora_Tq_R	ATTACTUGTACAGCTCGTCCATGC	62.1
*aceEF*_HR1_F	AGATCCUCGAAGACTCGCTTGAAGAGG	59.7
*aceEF*_HR1_R	AGCCATGUAAGCCAGCACACTGC	55.9
*aceEF*_HR2_F	ACATGGCUTGCTCCAGGG	58.3
*aceEF*_HR2_R	AGGTCGACUCGATGAACTGCTGGTTGCG	59.6
Seq-LP_F	ACCAACTTTTCCGCTTTGCAC	57.2
Seq-LP_R	CGAAAGACTGGGCCTTTCGT	58.4
*aceEF*_g-check_F	GTTTGGCTGGAGATTTTGGG	54.8
*aceEF*_g-check_R	CCTTGATCGGCGTGAAATAG	53.8
*aceEF*_F	GCGCGCTCATTCGCGTACCTGACAT	N.A.
*aceEF*_R	AAACATGTCAGGTACGCGAATGAGC	N.A.

The melting temperatures (T_m) were calculated using the *on line* Tm calculator by Thermo Fisher Scientific (only the annealing parts of the primers were included in the analysis). For primers used in PCR amplifications with *Phusion U* polymerase, '*Phusion* DNA polymerase' was chosen as the setting, with a primer concentration of 0.5 μM. For primers used for colony PCR, '*Taq*-based DNA polymerase' was chosen as the setting, with a primer concentration of 0.2 μM. N.A., not applicable.

Table S2 · Assembly of pGNW plasmids with genes encoding fluorescent proteins within a chromosomal landing pad.

**Fluorescent protein**	**Plasmid backbone** (oligonucleotide)	**Primers for HR1**	**Primers for gene insert**	**Primers for HR2**
msfGFP	**pGNW2** (pGNW_LP_F/ BCD2_R)	–	GFP_F/ GFP_R	–
mBFP2	**pGNW2** (pGNW_LP_F/ BCD2_R)	–	BFP2_F/ BFP2_R	–
mOrange2	**pGNW4** (pGNW-USER_F/ pGNW-USER_R)	LP_HR1_F/ BCD2_R	Ora_Tq_F/ Ora_Tq_R	pGNW_LP_F/ LP_HR2_R
mTurquoise2	**pGNW6** (pGNW-USER_F/ pGNW-USER_R)	LP_HR1_F/ BCD2_R	Ora_Tq_F/ Ora_Tq_R	pGNW_LP_F/ LP_HR2_R

Listed are the respective plasmid backbones used for each fluorescent protein, as well as the primers that were used to amplify these plasmids and the additional fragments to assemble the corresponding DNA fragments. The sequences of the primers are presented in Table S1.

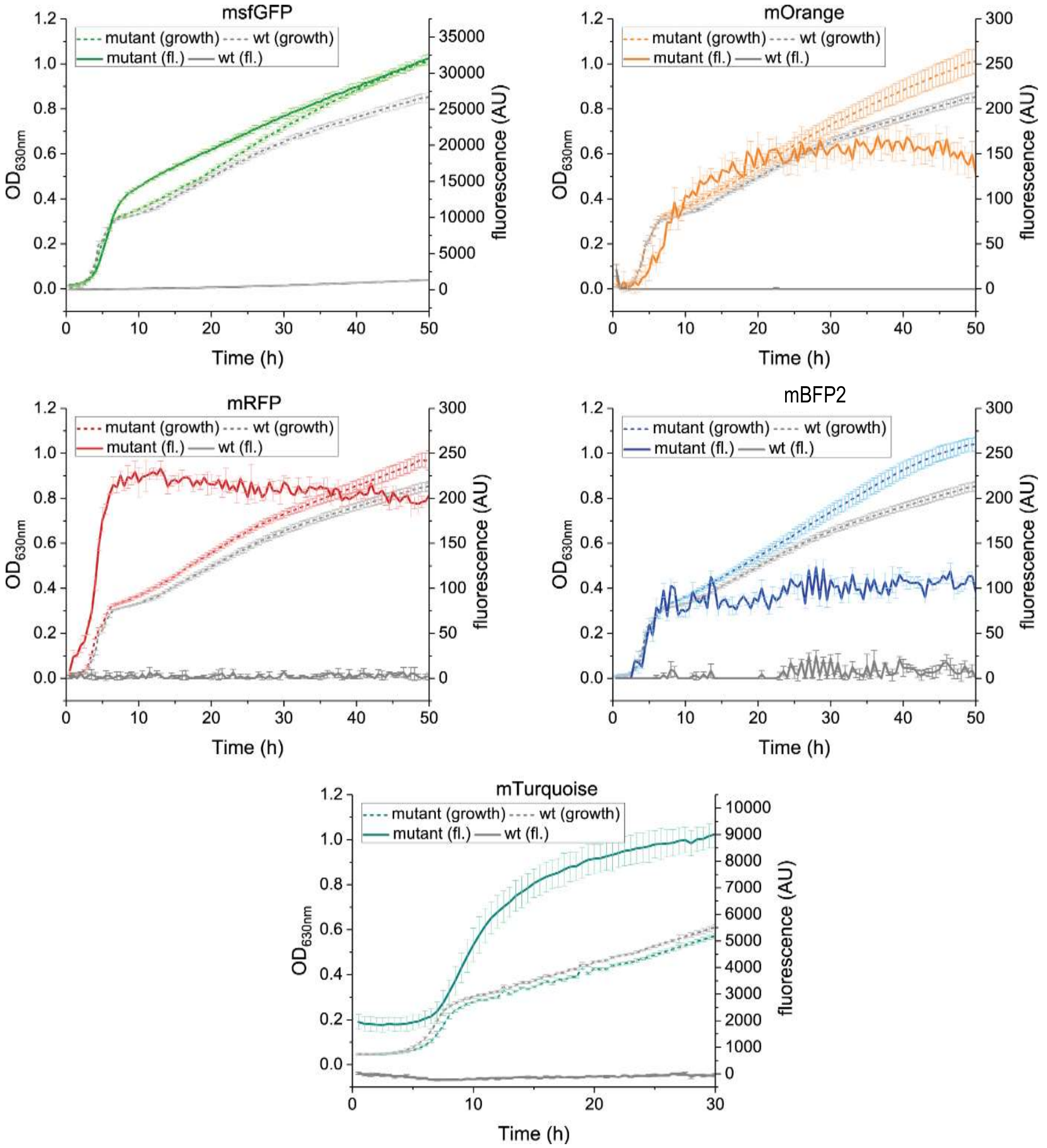

Figure S1 · Kinetics of the accumulation of selected fluorescent proteins measured in *P. putida* KT2440. Mutant and wild-type cells were grown in 96-well plates with de Bont medium supplemented with 30 mM citrate as the sole carbon source. Growth and fluorescence kinetics were acquired by measuring the culture optical density at 630 nm ($OD_{630\ nm}$) as well as the excitation/emission values of fluorescent proteins as follows: msfGFP at 485 nm/528 nm, mRFP1 at 582 nm/609 nm, mBFP2 at 385 nm/450 nm, mOrange2 at 541 nm/567 nm, and mTurquoise2 at 451 nm/477 nm. Error bars indicate standard deviations from at least three biological replicates.

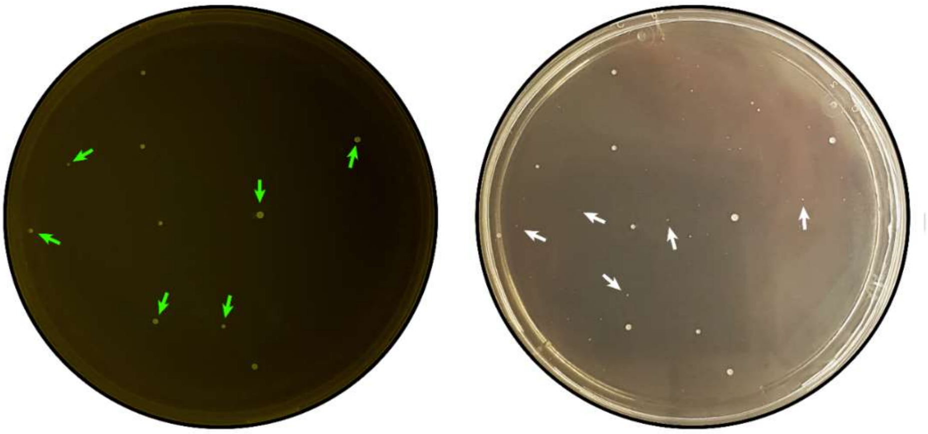

Figure S2 · Deletion of *aceEF* in *P. putida* KT2440 using a synthetic CRISPR-Cas9 device for counterselection. Green arrows in the left plate indicate fluorescent colonies that escaped restriction by Cas9; white arrows in the right plate identify colonies arising from *P. putida* Δ*aceEF* cells.
